# The neuroprotective potential of phytochemicals in traumatic brain injury: mechanistic insights and pharmacological implications

**DOI:** 10.3389/fphar.2023.1330098

**Published:** 2024-01-04

**Authors:** Gulam Mustafa Hasan, Saleha Anwar, Anas Shamsi, Sukhwinder Singh Sohal, Md. Imtaiyaz Hassan

**Affiliations:** ^1^ Department of Basic Medical Science, College of Medicine, Prince Sattam Bin Abdulaziz University, Al-Kharj, Saudi Arabia; ^2^ Centre for Interdisciplinary Research in Basic Sciences, Jamia Millia Islamia, New Delhi, India; ^3^ Centre of Medical and Bio-Allied Health Sciences Research, Ajman University, Ajman, United Arab Emirates; ^4^ Respiratory Translational Research Group, Department of Laboratory Medicine, School of Health Sciences, College of Health and Medicine, University of Tasmania, Launceston, TAS, Australia

**Keywords:** phytonutrients, traumatic brain injury, multi-targeted therapies, therapeutic intervention, secondary injury cascades (SIC), blood-brain barrier (BBB), neurodegenerative disorders (NDs)

## Abstract

Traumatic brain injury (TBI) leads to brain damage, comprising both immediate primary damage and a subsequent cascade of secondary injury mechanisms. The primary injury results in localized brain damage, while the secondary damage initiates inflammatory responses, followed by the disruption of the blood-brain barrier, infiltration of peripheral blood cells, brain edema, and the release of various immune mediators, including chemotactic factors and interleukins. TBI disrupts molecular signaling, cell structures, and functions. In addition to physical tissue damage, such as axonal injuries, contusions, and haemorrhages, TBI interferes with brain functioning, impacting cognition, decision-making, memory, attention, and speech capabilities. Despite a deep understanding of the pathophysiology of TBI, an intensive effort to evaluate the underlying mechanisms with effective therapeutic interventions is imperative to manage the repercussions of TBI. Studies have commenced to explore the potential of employing natural compounds as therapeutic interventions for TBI. These compounds are characterized by their low toxicity and limited interactions with conventional drugs. Moreover, many natural compounds demonstrate the capacity to target various aspects of the secondary injury process. While our understanding of the pathophysiology of TBI, there is an urgent need for effective therapeutic interventions to mitigate its consequences. Here, we aimed to summarize the mechanism of action and the role of phytochemicals against TBI progression. This review discusses the therapeutic implications of various phytonutrients and addresses primary and secondary consequences of TBI. In addition, we highlighted the roles of emerging phytochemicals as promising candidates for therapeutic intervention of TBI. The review highlights the neuroprotective roles of phytochemicals against TBI and the mechanistic approach. Furthermore, our efforts focused on the underlying mechanisms, providing a better understanding of the therapeutic potential of phytochemicals in TBI therapeutics.

## 1 Introduction

A traumatic brain injury (TBI) is an injury that takes place when an external force inflicts harm upon the brain. This force can arise from various situations, including falls, accidents, sports-related incidents, and physical violence (Maas et al., 2022). TBIs can exhibit a range of severities, encompassing mild to severe, and can result in lasting and significant effects on an individual’s physical, cognitive, emotional, and behavioral abilities. TBI can be categorized into two phases: first, the initial injury arising from the mechanical trauma, and second, the TBI-related consequences. The initial injury can result in the direct bruising of cranial tissue or the shearing of axons as the brain is rapidly compelled to change its position (Johnson et al., 2013a). Several secondary injury cascades (SICs) are commenced following the mechanical trauma. If left uncontrolled, these cascades result in further TBI-related pathology, leading to various neurological issues. The clinical symptoms of TBI correlate directly with the severity of the initial injury and the extent and duration of the SICs. The onset and scale of TBI-related SIC are highly intricate processes, resulting in a disturbance of the mechanisms that typically regulate multiple factors for normal brain functioning. These subsequent injuries involve the disturbance of the blood-brain barrier (BBB), excitotoxicity induced by glutamate, oxidative stress, and neuroinflammation, all of which can develop in a time-dependent manner following the initial mechanical injury (Chiu et al., 2016).

One key goal of therapeutic management after the trauma is to manage or reduce the progression of SIC. These events could potentially result in the salvation of nearby regions (known as the penumbra) and improve the chances of a favorable outcome. Researchers have explored the potential of substances possessing antioxidant properties as innovative therapeutic agents to reduce oxidative stress and decelerate cellular harm induced by reactive compounds. Antioxidants can either intervene in the generation of reactive oxygen species (ROS) within mitochondria or function as inhibitors of nicotinamide adenine dinucleotide phosphate (NADP). These mechanisms can potentially reduce secondary injuries and enhance clinical outcomes (Fernández-Gajardo, Matamala, Carrasco, Gutiérrez, Melo and Rodrigo, 2014).

Many naturally occurring substances exhibit antioxidant properties (Alam et al., 2022a; Ali et al., 2022; Alam et al., 2022b; Alam et al., 2022c). Their antioxidant mechanisms are linked to their influence on the pathways and enzymes within mitochondria. Utilizing substances with antioxidant properties can decelerate the degeneration of the brain and influence the outcome or the intensity of the complications. Increasing evidence suggests that neuroinflammation and oxidative stress play a significant role in contributing to neurological impairments and the development of posttraumatic epileptogenesis, in addition to cytotoxicity and damage to neurons and glial cells (Shin et al., 2011; Battaglini et al., 2021; Fatima et al., 2022a; Fatima et al., 2022b; Alam S. et al, 2022d; Anwar et al., 2023).

Over the years, researchers have investigated different facets of TBI using various animal models to gain deeper insights into its pathophysiology and explore potential treatment options. Various TBI models have been used to study the progression of the disease and the role of multiple modulators of TBI. The promising role of phytochemicals in TBI treatment offers an exciting avenue for research and clinical applications. The future holds great potential for personalized, multi-targeted therapies, innovative delivery systems, and a more comprehensive understanding of TBI, ultimately leading to improved outcomes and quality of life for TBI patients.

In the review, we have summarized the pathophysiology of TBI, mechanisms associated with TBI progression, and the role of phytochemicals as neuroprotective agents in TBI. Various mechanisms, such as glutamate-induced excitotoxicity, mitochondrial dysfunction, neurotoxicity, etc., are associated with the consequences of TBI. Phytochemicals have emerged as a neuroprotective agent in TBI by acting against these consequences. This review further highlights the role of different phytochemicals and the mechanical approach associated with the phytochemical to exhibit the neuroprotective effect in TBI.

## 2 Pathophysiology of traumatic brain injury

In the United States, TBI accounts for around 40% of deaths resulting from acute injuries and stands as the primary cause of death in individuals under the age of 45. Moreover, TBI imposes a significant financial burden, with an estimated total annual cost of $37.6 billion, of which $12.7 billion represents lost income due to premature death (Werner and Engelhard, 2007; Popescu, Anghelescu, Daia and Onose, 2015). TBI results from external mechanical forces that can cause temporary or permanent physical, psychological, or cognitive impairments, often accompanied by an altered state of consciousness (Zhao et al., 2011; Maxwell, 2012). Symptoms of TBI encompass dizziness, headaches, amnesia (forgetfulness), and nausea, which may show improvement over days to weeks following the injury. However, in cases of severe injury, long-term behavioral and cognitive impairments can result (Yamamoto, Levin and Prough, 2018; Thapa, Khan, Singh and Kaur, 2021). There are fragmented pieces of evidence indicating a higher occurrence of neurodegenerative disorders (NDs) like Alzheimer’s disease (AD), Parkinson’s disease (PD), and chronic traumatic encephalopathy because of head trauma (Safinia et al., 2016). TBI treatment includes pharmacotherapy, cognitive therapies and surgeries, which depend on the severity of the condition (Stocchetti et al., 2017).

TBI classification involves several scales and metrics. For instance, the Glasgow Coma Scale (GCS), along with the duration of altered levels of consciousness (LOC) and post-traumatic amnesia (PTA), serve as valuable tools to evaluate the clinical severity of TBI (Mehta and Chinthapalli, 2019; Bodien et al., 2021). Individuals who experience severe TBI face an elevated risk of secondary brain injury, which can result in adverse outcomes such as disability, a vegetative state, or even death. Additional classification is based on the regions of injury: focal injury encompasses mass lesions like contusions, subdural hematoma, epidural hematoma, and intra-parenchymal haemorrhage, while diffuse injury involves axonal, hypoxic-ischemic and micro-vascular injuries (Leitgeb et al., 2012). As a result of brain trauma, TBI can be categorized into primary and secondary brain injuries, depending on the outcome (Mckee and Daneshvar, 2015).

Primary brain injury results in brain damage, reduced oxygen supply, and extensive tissue necrosis, affecting all cellular components and blood vessels within the brain. Neuronal and glial cell death in the necrotic region is linked to impaired blood flow and the disruption of the blood-brain barrier (BBB) integrity, potentially resulting in additional intracerebral haemorrhage and edema due to vascular leakage (Corps, Roth and McGavern, 2015). The astroglial syncytial systems, assisted by aquaporin-4 (AQP-4), facilitate the removal of excessive edema fluid from the brain (Rash, Davidson, Yasumura and Furman, 2004). The intensity of post-injury inflammation assisted with pro-inflammatory cytokines contributes to blood vessel damage (Kwiecien et al., 2020).

**FIGURE 1:**
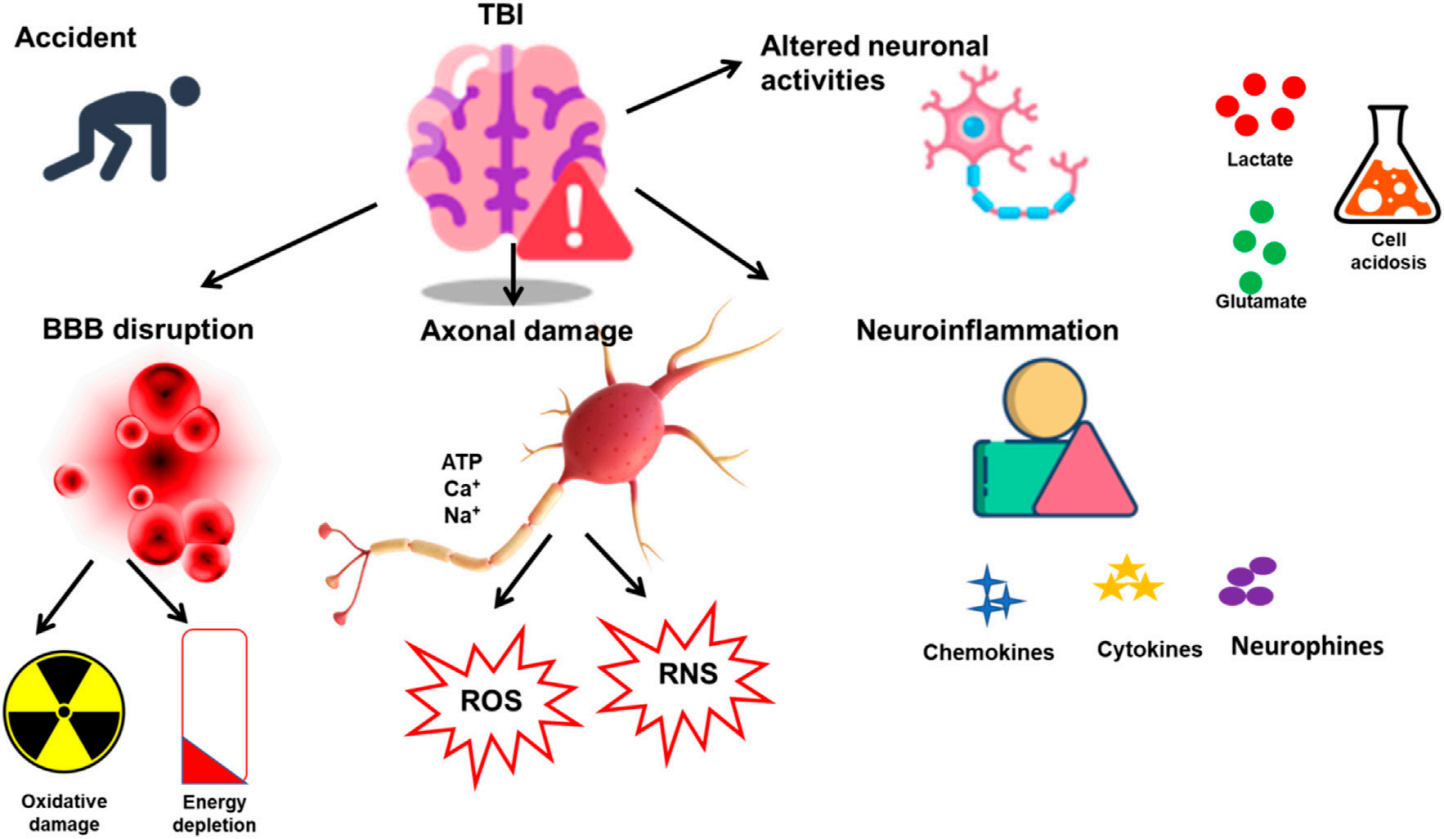
SICs associated with TBI. TBI is induced in response to sudden damage to the neurons and brain. Various SICs are related to the onset of TBI, such as BBB disruption, neuronal damage, and neuroinflammation. The SICs further result in alterations in various cellular functions such as oxidative damage, ROS and RNS release, neuroinflammatory agents, and cell acidosis.

TBIs can further be classified as penetrating and closed-head injuries. In penetrating injuries, the object penetrates both the skull and the dura, resulting in direct injury to the brain, while in closed-head injuries, the skull and dura remain intact. TBIs are categorized based on severity as mild, moderate, and severe based on clinical parameters such as loss of consciousness, amnesia, neurological symptoms, and the findings from structural brain imaging, such as CT or MRI scans (Blyth and Bazarian, 2010). Moderate and severe TBIs require neurosurgical interventions and intensive care. In contrast, mild TBI and concussion are synonymous terms to describe the mildest form of TBIs, comprising 80%–90% of cases [4–6]. Mild TBI usually stems from non-penetrating blunt head trauma, leading to temporary symptoms identified through clinical assessments, patient self-reporting, or witness observations (if present). Symptoms can vary widely and encompass physical indications (such as nausea, vomiting, dizziness, and headaches), cognitive manifestations (like difficulties in concentration and memory), behavioral changes (including irritability and emotional instability), and the potential for loss of consciousness [5]. TBI is further associated with secondary injury cascades through various mechanisms. Various factors such as excitotoxicity, oxidative stress, and others play a significant role in the SICs. Some of them are discussed in the following paragraphs. Figure 1 shows the pathophysiology and SICs associated with TBI.

## 3 Excitotoxicity in TBI

The disruption of the BBB resulting from TBI leads to the release of excessive neurotransmitters and a breakdown in the normal glutamate reuptake by transporters (Chamoun, Suki, Gopinath, Goodman and Robertson, 2010). Glutamate and its various byproducts bind and activate the ionotropic (IT) and metabotropic (MT) types of glutamate receptors. IT glutamate receptors, such as NMDA and AMPA receptors, permit the entry of sodium, potassium, and calcium ions, causing membrane depolarization (Brustovetsky, Bolshakov and Brustovetsky, 2010). In TBI, the excessive glutamate release leads to an upregulation of these receptors, disrupting ion balance by allowing extracellular calcium and sodium ions into the cells (Meldrum, 2000). It has been observed that the GluN2B subunit, present in synaptic cytosol, plays a role in mediating the excitotoxic response (Wyllie, Livesey and Hardingham, 2013). Elevated intracellular calcium levels trigger downstream signalling molecules like Ca2+/calmodulin-dependent protein kinase II (CAMK II), protein kinase C (PKC), mitogen-activated protein kinases (MAPK), and protein phosphatases (PPs).

The excessive cytosolic calcium activates apoptotic proteins such as calpain, calcineurin, and caspases, ultimately leading to cell death (Folkerts, Parks, Dedman, Kaetzel, Lyeth and Berman, 2007; Weber, 2012). Excitatory neurotransmitters cause cells to succumb to oxidative stress, resulting in excitotoxic cell death (Chamoun et al., 2010). Shortly after the trauma, the shearing and stretching forces resulting from the head injury induce glutamate-independent excitotoxicity through NMDA receptor activation (LaPlaca and Thibault, 1998). Various studies have identified the GluN2B subunit as a mediator of mechanosensitive responses (P. Singh et al., 2012). Various phytochemicals have shown protective effects against glutamate-induced excitotoxicity and are summarized in Table 1.

**TABLE 1:** Phytochemicals against glutamate-induced excitotoxicity.

Phytochemical	Methodology	Results	Reference
Barberini	A. Protective effect of the compound against glutamate excitotoxicity was studied on PC12 and N2a cells	1. Reduced ROS	Anbai et al. (2022); Chen et al. (2014), Huang et al. (2018), Sadeghnia et al. (2017); Wang and Zhang (2018); Lin et al. (2013)
		2. Reduced Malondialdehyde (MDA)	
		3. DNA fragmentation	
		4. Elevated superoxide dismutase (SOD) activity	
		5. Decreased levels of caspase-3	
		6. Decreased	
	B. Release of glutamate from rat’s cortical synaptosomes was studied	Bax/Bcl-2 ratio	
		1. Inhibition of the release of glutamate responsible for excitotoxicity	
		2. Chelation of extracellular Ca^2+^ ions	
Cinnamaldehyde (CA)	CA was studied for its neuroprotective effects on glutamate-mediated excitotoxicity. CA pre-treated PC12 cells were examined	1. Reduced cell death	Deng et al. (2022), Lv et al. (2017)
		2. Reduced ROS production	
		3. Reduced caspase 3/9	
		4. Inhibition of release of cytochrome C	
Curcumin	A. Endogenous glutamate release was monitored in nerve terminals of the rat prefrontal cortex	Inhibition of glutamate release from rat prefrontocortical synaptosomes	Chang et al. (2014); Lin et al. (2011) Chang et al. (2014)
	B. The effect of curcumin was studied in rat pheochromocytoma PC12 cell lines	1. Upregulated glutathione peroxidase 1, glutathione disulphide and Ca^2+^ influx, 2. Downregulated glutathione, glutathione reductase, SOD and catalase	
		3. Increased cell apoptosis	
Huperzine A (HupA)	HupA was studied for its effects on glutamate toxicity in hippocampal HT22 cells derived from mice	Reduced oxidative glutamate excitotoxicity	Mao et al. (2016)
Naringenin (NAG)	Neuroprotective impacts of NAG on glutamate-induced excitotoxicity in hippocampal neurons of neonatal mice	1. Increased phosphorylations of ERK1/2 and Akt	Nouri et al. (2019); X.-H. Xu et al. (2015); Xue, et al. (2019)
		2. Release of BDNF and neuroprotective cytokines	
		3. Neuroprotection	
Protopanaxadiol	The neuroprotective effects of the compound were studied in PC12 cells	1. Prevention of glutamate-mediated apoptosis	Bak et al. (2016)
	Excitotoxicity was induced by glutamate (5 mM)	2. Enhanced mitochondrial function	
		3. Increased anti-oxidant activity	
Thymoquinone (TQ)	The protective effects of TQ were studied against glutamate-induced cell death in SH-SY5Y neuronal cells	1. Reduced glutamate-induced ROS generation and mitochondrial dysfunction	Al Mamun et al. (2015); Gülşen et al. (2016); Farkhondeh et al. (2018); Fouad et al. (2018)

## 4 Mitochondrial dysfunction

Mitochondrial dysfunction is a prominent event of TBI, resulting in disruptions to physiological and metabolic processes and ultimately culminating in cell death. The increased entry of calcium ions (Ca2+) into mitochondria can lead to ROS generation and depolarization of the mitochondrial membrane, all occurring without ATP synthesis (Susin et al., 1998; Xiong et al., 1999). Consequently, the electron transport chain (ETC) and oxidative phosphorylation (OXPHOS) disrupt calcium regulation and metabolic function (Naga et al., 2007). Mitochondrial dysfunction additionally leads to a structural alteration in the adenine nucleotide translocator protein when it interacts with Cyclophilin D. Mitochondrial damage results in oxidative stress, followed by apoptosis and reduced cellular energy production. These changes in brain cells impair neurological functions, evident in individuals suffering from TBI. The intricate mitochondrial dysfunction following TBI necessitates targeted treatment to address the secondary injury. Phytochemicals, naturally occurring compounds found in various plant sources, have gained attention for their potential therapeutic role in treating TBI by targeting mitochondrial dysfunction. Astaxanthin (AXT), a carotenoid compound, is utilized as a dietary supplement (Hussein et al., , 2006). AXT was studied in mouse neuronal cells (HT22) with glutamate toxicity at doses 1.25–5 µM. AXT showed various effects on HT22 cells, including attenuating mitochondrial dysfunction by regulating the Akt/glycogen synthase kinase (GSK)-3β signaling pathway. Barberine has also shown neuroprotective effects via elevating the mitochondrial membrane potential (MMP) (Moneim, 2015).

Curcumin has also shown inhibitory effects on the mitochondrial dysfunction in PC12 cells, reducing ROS production and neurotoxicity (R. Wang et al., 2008). Protopanaxadiol is a natural compound that prevents glutamate-induced toxicity by improving mitochondrial function and enhancing anti-oxidant activity (Bak et al., 2016). Tanshinone IIA also suppresses mitochondrial dysfunction by modulating the MAPK pathways in the neuroblastoma cell line (Li et al., 2017).

## 5 Oxidative stress

Elevated levels of free radicals, encompassing both reactive nitrogen species (RNS) and reactive oxygen species (ROS), may arise due to secondary cell death and oxidative stress. Excessive production of ROS disturbs mitochondrial function, damaging the mitochondrial membrane through lipid peroxidation (Shohami and Kohen, 2011). In response to the damaged cells after TBI, there is an increased ROS generation from the electron transport chain (ETC). Conversely, after TBI, the accumulation of Ca2+ facilitates nitric oxide (NO) generation by nitric oxide synthases (NOS) (Deng et al., 2007). Moreover, the continuous release of ROS and lipid peroxidation unfavourable influences cerebral blood flow, leading to immunosuppression and brain plasticity (Shohami and Kohen, 2011). Table 2 summarizes some phytochemicals associated with reducing oxidative stress in neurological cell lines and TBI models.

**TABLE 2:** Neuroprotective role of phytochemicals via management of oxidative stress.

Compound	Methodology	Results	References
Apocynin	Mice and rats were administered varying doses of apocynin, ranging from 4 to 100 mg/kg, through the intraperitoneal (i.p.) route for pretreatment. Post-trauma interventions in mice involved an i.p. dose of 5 mg/kg or a range of 0.05–5 mg/kg administered subcutaneously (s.c.).	1. Inhibit NOX activity upregulated following TBI	Ferreira et al. (2013); Loane, Stoica, Byrnes, Jeong and Faden. (2013)
		2. Decrease of oxidative stress	
Caffeic acid phenethyl ester (CAPE)	Adult male rats received CAPE (10 μmol/kg) intraperitoneally (i.p.) immediately following a head injury	1. Reduction in lipid peroxidation	(Manzar Alam et al., 2022; Kerman et al. (2012); Pérez et al. (2023)
		2. Enhancement of essential antioxidants	
Curcumin	Adult rats underwent a mild injury and were fed a diet containing curcumin (500 ppm) for a 4-week pretreatment period or received post-treatment for 2 weeks	The 4-week pretreatment resulted in a reduction of oxidative stress, an increase in BDNF levels, and the preservation of synaptic proteins	Sharma et al. (2010); Sharma et al. (2009); Wu et al. (2006); Wu et al. (2011)
Formononetin (fp)	Following a closed head injury, FN treatment was administered intraperitoneally (i.p.) at 10 or 20 mg/kg doses, starting 5 days after the TBI.	Increased antioxidant enzymes (GPx and SOD) and decreased lipid peroxidation (LP)	Li et al. (2014); Singh et al. (2023)
Ginseng	Two weeks after a significant injury, the subjects orally consumed 100 or 200 mg/kg of ginseng	Eliminated oxidative stress and neuroinflammation in adult rats with injury using a modified Marmarou injury model	Kumar et al. (2014); Lee and Kim. (2023)
Hydroxysafflor yellow A (HSYA)	The animals received pretreatment with HYSA intravenously (i.v.) 30 min before and 6 h after the injury	HSYA treatment significantly improved mitochondrial function, boosting antioxidant activity and reducing lipid peroxidation (LP)	Bie et al. (2010); Lai et al. (2023)
Luteolin	A modified weight-drop closed head injury model was utilized; animals received treatment with luteolin at doses of 10, 30, or 50 mg/kg intraperitoneally (i.p.) 30 min after the trauma	Decrease in oxidative stress and an elevation in the antioxidant GPx levels	Kempuraj et al. (2021); Xu et al. (2014)
Pycnogenol (PYC)	Young adult rats experienced a moderate unilateral CCI injury. They were then promptly treated with PYC (100 mg/kg, intraperitoneal), followed by two additional doses administered 3 and 6 h after the injury	She has elevated several antioxidants (GSH, GPx, and catalase) while concurrently reducing oxidative stress markers	Scheff et al. (2013); Ozoner et al. 2019; Çolak et al. (2021)

## 6 Neuroinflammation

TBI triggers a complex array of immune and inflammatory responses in tissues, resembling the reactions observed in ischemia/reperfusion injury. The process activates cellular mediators of inflammation, such as prostaglandins and free radicals (Werner and Engelhard, 2007; Thapa et al., 2021). Analysis of cerebrospinal fluid and post-mortem brain tissue from individuals who suffered TBIs (Frugier et al., 2010; Goodman et al., 2009) and experiments conducted on rodents (Lotocki et al., 2009; Semple et al., 2010) indicated that within 24 h of the trauma, polymorphonuclear leukocytes and cytokines released inflammatory agents like IL-6, IL-1β, and TNF-α. The persistent secretion of cytokines indicates disruptions in the blood-brain barrier (BBB), resulting in edema and neurological impairments. TNF-α, belonging to the Fas family, exhibits a robust interaction with the Fas ligand and triggers caspases, initiating programmed cell death (Bye et al., 2007).

Moreover, endothelial and leukocyte cell adhesion molecules like ICAM-1 and VCAM-1 promote the recruitment of leukocytes and immune cells to the site of injury through their interactions with endothelial cells (Frugier et al., 2010). In cases of prolonged neuroinflammation, macrophages activate microglial cells, leading to an increased release of astrocytes, a phenomenon observed in survivors of TBI for many years following the initial injury (Johnson et al., 2013b). The involvement of GSK-3β in the physiological framework of mild traumatic brain injury (mTBI) has been investigated at both the cellular and behavioral dimensions (Shapira et al., 2007).

The reduction of neuroinflammation in TBI through phytochemicals is a subject of growing interest in neurology and medicine. Research suggests that phytochemicals can modulate the release of proinflammatory cytokines, such as TNF-α and IL-6, and reduce oxidative stress, ultimately contributing to TBI neuroinflammation. Various phytochemicals have been studied for their inhibitory effects on neuroinflammation. Curcumin mitigated the neurotoxic effects induced by glutamate through the inhibition of endoplasmic reticulum stress-associated activation of TXNIP/NLRP3 inflammasome in a manner that relied on 5’ AMP-activated protein kinase, as documented in reference (Feeney et al., 1981). Additionally, Lovastatin was associated with a decrease in the expression of proinflammatory cytokines, specifically TNF-α and IL-1β. A summarized neuro score demonstrated significant improvement during the initial 7 days.

In an experimental study, a pretreatment approach was employed to assess lovastatin’s potential as a neuroprotective agent in the context of a subsequent moderate TBI (CCI-induced TBI), as described in reference (S.-F. Chen et al., 2007). Adult rats received lovastatin injections (4 mg/kg, intraperitoneal) for 5 days preceding a unilateral injury focused on the bregma region. 6 h after the traumatic event, there was a notable decrease in the messenger RNA (mRNA) levels of TNF-α and IL-1β. 4 days after the injury, the researchers observed a significant decrease in the contusion volume, accompanied by a reduction in FJB staining.

The reduction of neuroinflammation in TBI through phytochemicals is a subject of growing interest in neurology and medicine. Research suggests that phytochemicals can modulate the release of proinflammatory cytokines, such as TNF-α and IL-6, and reduce oxidative stress, ultimately contributing to TBI neuroinflammation. Various phytochemicals have been studied for their inhibitory effects on neuroinflammation. Curcumin mitigated the neurotoxic effects induced by glutamate through the inhibition of endoplasmic reticulum stress-associated activation of TXNIP/NLRP3 inflammasome in a manner that relied on 5’ AMP-activated protein kinase, as documented in reference (Feeney et al., 1981).

## 7 BBB breakdown

While it is technically considered part of the primary injury, the breach of the blood-brain barrier (BBB) plays a role in the development of brain edema, which is a critical early element of secondary injury cascades (SIC) associated with various forms of brain injury (Kelley et al., 2007; Das et al., 2012; Mendes Arent et al., 2014). Cerebral edema is characterized by an expansion in brain tissue volume resulting from the buildup of fluid, and it occurs through two fundamental mechanisms: vasogenic and cytotoxic (Vink and Nimmo, 2009; Hinson et al.,, 2015). Vasogenic edema arises due to heightened blood-brain barrier (BBB) permeability, leading to an imbalance in the oncotic and hydrostatic pressures that control the movement of fluids between the bloodstream and the brain interstitial space (Kimelberg, 1995). An intact blood-brain barrier (BBB) prevents the diffusion of water-soluble molecules exceeding 500 Da. However, once the BBB is compromised, brain-specific proteins can be observed in the bloodstream and cerebrospinal fluid (CSF) (Reiber and Peter, 2001; Stahel et al., 2001; Blyth et al., 2009). Cytotoxic edema is marked by the swelling of brain structural cells within the intracellular space, and this occurs while the blood-brain barrier remains unbroken. This edema primarily manifests in grey matter due to energy depletion and insufficient blood supply. Regarding its mechanism, cytotoxic edema is linked to malfunctioning ATPase-dependent cellular pumps and water buildup within the cells among osmotically active solutes (Unterberg et al., 2004; Stiefel et al., 2005). Elevated glutamate levels can play a role in promoting cytotoxic edema by raising intracellular sodium levels and facilitating the movement of water into the cells, subsequently increasing the volume of intracellular fluid (Duvdevani et al., 1995; Floyd et al, 2005; Yi and Hazell, 2006).

In a recent investigation, the application of Baicalein was examined using a subarachnoid haemorrhage model, a condition frequently observed following TBIs, which may hold significance for advancing innovative therapeutic strategies. Treatment was initiated 30 min after the injury with 30 mg/kg or 100 mg/kg of Baicalein. This treatment regimen led to a reduction in blood-brain barrier (BBB) permeability, edema, and neuronal apoptosis, ultimately resulting in an enhancement of the subjects’ neuroscores (Chen et al., 2008; Kerman et al., 2012; Wang et al., 2015). In a separate investigation, adult male rats were exposed to mild to moderate TBI induced by controlled cortical impact (CCI). These rats were then administered CAPE therapy at 10 mg/kg via intraperitoneal injection, commencing 30 min after the injury.

**FIGURE 2:**
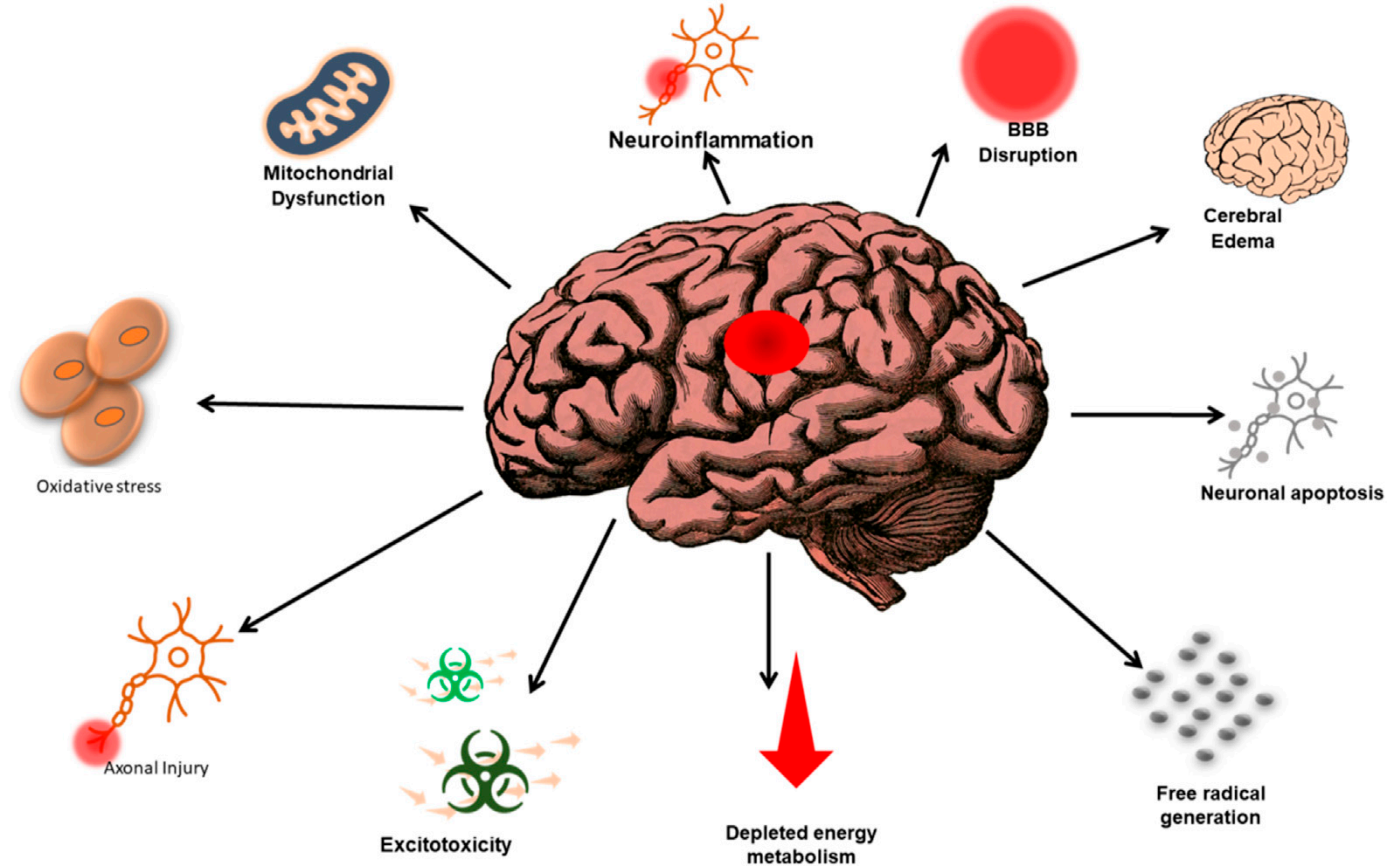
Representation of the complex relationship between the various factors that lead to TBI and the subsequent consequences associated with TBI-related events. The annotations aim to enhance understanding and awareness of the multifaceted nature of TBI and its impacts.

In some cases, the treatment was extended for an additional 4 days. When assessed 24 h after a single treatment, the rats displayed decreased blood-brain barrier (BBB) permeability and a decrease in the loss of cortical cells (Kerman et al., 2012; Zhao et al., 2012). In a recent study, the potential therapeutic benefits of EA were examined following a mild to moderate diffuse injury, explicitly using the Marmarou model, in young adult rats. These animals received a pretreatment regimen for 7 days through oral administration of EA at a dose of 100 mg/kg. This pre-treatment approach reduced BBB permeability (Marmarou et al., 1994). Figure 2 shows various factors leading to TBI.

## 8 Role of phytochemicals in TBI

TBI is a significant public health concern, with over 2.8 million new cases annually in the United States alone (Taylor et al., 2017; Horstemeyer et al., 2019). TBI occurs when an external mechanical force impacts the head, leading to temporary or permanent impairments in brain function (Jenkins, 2023). TBI could be of forms, i.e., primary and secondary phases of damages (Ye et al., 2023). The primary injury induced immediately by the external force causes tissue damage through skull fractures, haemorrhages, and vascular injury (Xiong et al., 2013). However, the subsequent secondary injury arising minutes to months after the initial insult exacerbates the neuropathology through various cellular processes, including neuroinflammation, oxidative stress, metabolic dysfunction, and excitotoxicity (Walker and Tesco, 2013). This secondary phase offers a window for therapeutic interventions to mitigate further neuronal damage and improve outcomes. Currently, no FDA-approved drugs target the secondary neurodegenerative cascades induced by TBI (Prins and Matsumoto, 2014).

Therefore, research has focused on identifying neuroprotective agents from natural sources, such as dietary phytochemicals, which we will discuss in the later sections of this literature. Phytochemicals from fruits, vegetables, spices, teas, and herbs demonstrate therapeutic efficacy in preclinical TBI models through their anti-inflammatory, antioxidant, and anti-apoptotic properties. However, most findings arise from *in vitro* studies, warranting further research through *in vivo* models and clinical trials to validate their neuroprotective effects and mechanisms. The pathological changes could also include altered brain metabolism, excitotoxicity, free radical generation, neuroinflammation, blood-brain barrier dysfunction, and apoptosis. Phytochemicals derived from dietary sources can mitigate secondary brain injury by activating endogenous neuroprotective mechanisms.

During the secondary TBI phase, impaired mitochondrial function reduces ATP production, disrupting ionic homeostasis and promoting anaerobic metabolism. This results in lactic acid accumulation, cytokine release, and free radical generation (Hakiminia et al., 2022). Excitotoxicity also ensues, whereby excess extracellular glutamate overactivates NMDA receptors, increasing intracellular calcium levels and triggering protease activation and nitric oxide production (Engin and Engin, 2021).

Furthermore, oxidative stress arises from mitochondrial dysfunction and inflammation (Khan et al., 2022). This damages lipids, proteins, and nucleic acids. Lastly, activated microglia release proinflammatory cytokines like TNF-alpha and IL-1beta, inducing leukocyte infiltration, edema, and neurotoxicity (Jarrahi et al., 2020). Phytochemicals can modulate these secondary injury mechanisms through their anti-inflammatory, antioxidant, and anti-apoptotic properties (Al-Haj et al., 2022). For instance, curcumin inhibits TNF-alpha release from glial cells. Resveratrol reduces IL-1beta and other cytokines by inhibiting NF-kappaB signaling. Anthocyanins suppress microglial activation and subsequent neuroinflammation. Curcumin and anthocyanins also attenuate oxidative damage by scavenging free radicals and boosting endogenous antioxidants like glutathione (Pandey et al., 2022). Additionally, ginsenosides inhibit glutamate excitotoxicity by modulating NMDA receptor signaling and calcium fluxes.

**FIGURE 3:**
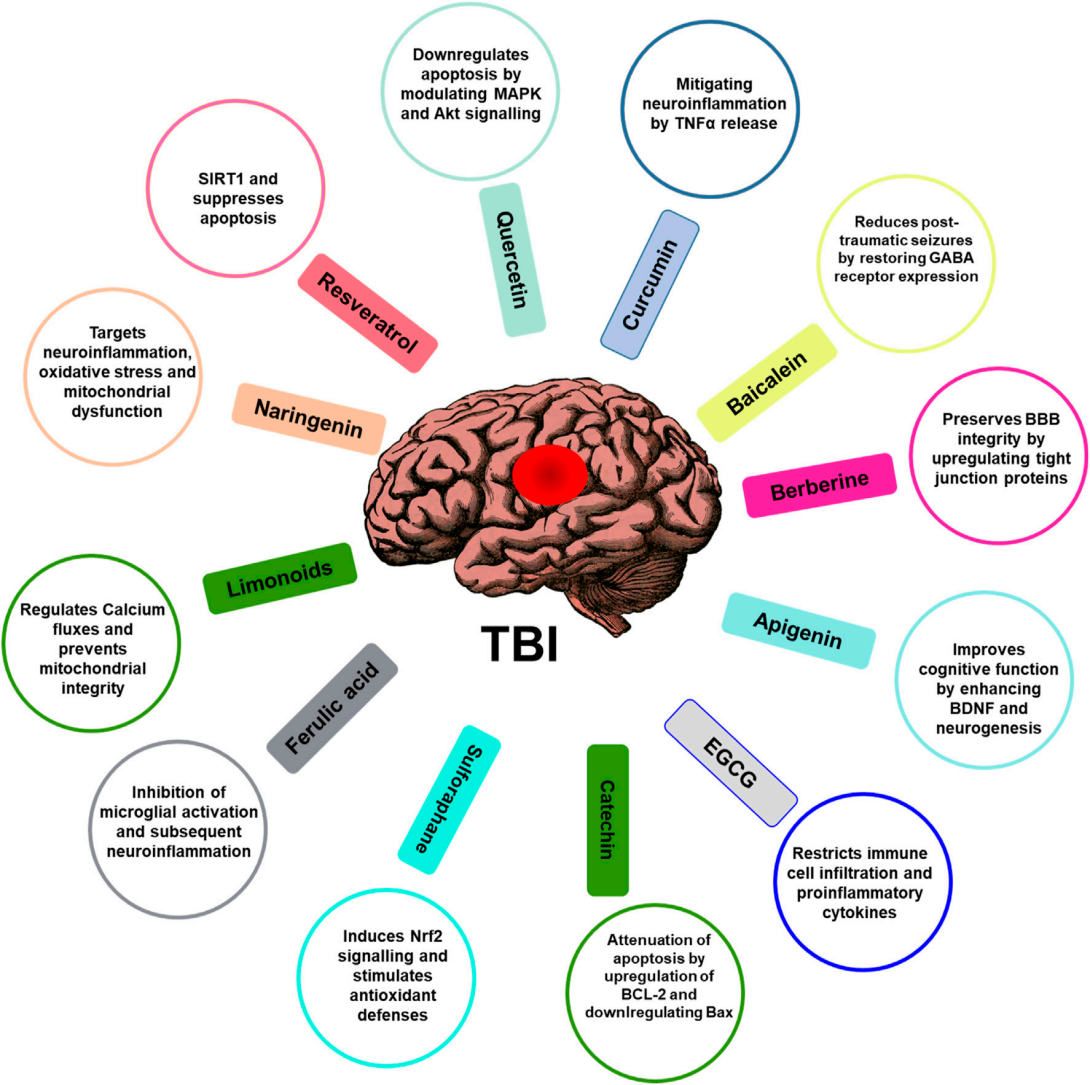
Role of phytochemicals in attenuating TBI. Phytochemicals and their mechanism of action associated with the function are illustrated. The figure illustrates phytochemicals such as flavonoids, polyphenols, alkaloids, or other compounds found in plants known for their potential neuroprotective properties in the therapeutic management of TBI and their possible mechanisms of action.

By mitigating secondary injury processes, phytochemicals can salvage viable neurons and improve neurological outcomes after TBI. In conclusion, phytochemicals activate cytoprotective mechanisms that can potentially prevent the progression of secondary brain damage following TBI. While no FDA-approved drugs currently target TBI pathogenesis, phytochemicals are promising therapeutic candidates warranting further investigation through rigorous *in vivo* and clinical studies. Optimizing one’s dietary phytochemical intake may help mitigate secondary neurodegeneration and impairment after a TBI. Figure 3 shows some of the phytochemicals as therapeutic agents in TBI management and their mechanism of action.

### 8.1 Resveratrol and its neuroprotective effects in TBI

Resveratrol (3,5,4′-trihydroxy-trans-stilbene) is a natural polyphenol in various plants such as grapes, peanuts, and berries (Ahmed et al., 2017). Several preclinical studies have elucidated the ameliorative effects of resveratrol against TBI-induced neuronal damage and cognitive impairments. Resveratrol remarkably attenuates neuronal apoptosis triggered by TBI. In a mouse model, resveratrol pretreatment conferred antiapoptotic effects by downregulating Bax, upregulating Bcl-2, and inhibiting caspase-3 activation (Xu et al., 2019).

The ability of resveratrol to modulate sirtuin-1 (SIRT1) and suppress acetylation of p53, a pro-apoptotic protein, has also been evidenced (Sin et al., 2014). A recent study showed that resveratrol pretreatment prevented neuronal apoptosis by reducing the expression of miR-21 through SIRT1 activation in a rat model of TBI (Kaminska et al., 2016). Resveratrol exhibits antioxidative properties that aid in combating TBI-induced oxidative stress. Administration of resveratrol suppresses malondialdehyde (MDA) and nitric oxide (NO) levels and restores glutathione (GSH) levels and superoxide dismutase (SOD) activity (Zhang et al., 2019). The Nrf2/HO-1 signaling cascade is also activated by resveratrol to confer neuroprotection against oxidative damage.

Neuroinflammation is another hallmark of secondary injury after TBI. Resveratrol attenuates the expression of proinflammatory mediators like IL-1β, IL-6, TNF-α, iNOS, and COX-2 (Rashad et al., 2018). The NF-κB pathway is also inhibited by resveratrol, thus reducing inflammation. In a mouse model, resveratrol suppressed neutrophil infiltration, microglia activation, and cytokine production induced by TBI (Phillips and Fahimi, 2018). In preclinical studies, resveratrol exhibits pleiotropic effects against TBI-induced neuronal apoptosis, oxidative stress, and neuroinflammation. Given its high bioavailability and ability to cross the blood-brain barrier, resveratrol is a potential adjuvant therapeutic for ameliorating secondary brain damage after TBI. However, further clinical trials are necessitated to establish its efficacy and safety for clinical use.

### 8.2 Quercetin and its ameliorating effects against TBI

Quercetin (3,3′,4′,5,7-pentahydroxyflavone) is a ubiquitous bioactive plant flavonoid in fruits, vegetables, leaves, seeds, and grains having numerous health benefits (Yousuf et al., 2020; Waseem et al., 2022). Emerging preclinical evidence indicates the neuroprotective effects of quercetin against neuronal damage associated with TBI. Quercetin treatment remarkably attenuates apoptotic cell death after TBI by modulating critical proteins involved in the mitochondrial apoptotic pathway. Studies report downregulation of Bax, cytochrome c, cleaved caspase-3, and upregulation of Bcl-2 levels upon quercetin administration in rodent models.

Quercetin also reduces neuronal apoptosis by inhibiting MAPK signaling and restoring PI3K/Akt pathway (Grewal et al., 2021). Furthermore, quercetin’s antioxidative activity facilitates oxidative stress-mediated secondary damage post-TBI. Quercetin treatment decreases malondialdehyde (MDA) and 8-iso-prostaglandin F2α (8-iso-PGF2α) levels and increases antioxidant enzymes like SOD and glutathione peroxidase (GPx) (Moslemi et al., 2022). Quercetin also exhibited free radical scavenging capacity by elevating glutathione (GSH) levels in the injured rat brain (Ansari et al., 2008). Neuroinflammation is a crucial contributor to TBI-induced neuropathology. Quercetin is evidenced to mitigate glial activation, inhibit production of pro-inflammatory cytokines like TNF-α, IL-1β, and IL-6, and attenuate NF-κB signaling after TBI. Moreover, quercetin reduces leukocyte rolling and adhesion to brain microvessels, indicating its anti-inflammatory effects (Shen et al., 2019). In TBI models, quercetin treatment confers multifaceted neuroprotection against oxidative stress, neuroinflammation, and apoptosis. However, clinical trials are warranted to translate the promising preclinical findings into viable therapeutic approaches for TBI patients.

### 8.3 Curcumin and its pleiotropic effects against TBI

Curcumin, a polyphenol derived from turmeric, has emerged as a potential neuroprotective phytochemical for TBI owing to its multifaceted effects. Studies demonstrate the potent antioxidant capacity of curcumin by mitigating oxidative damage and restoring the endogenous antioxidant systems after TBI. Curcumin remarkably reduces malondialdehyde (MDA) and nitrotyrosine levels and induces superoxide dismutase (SOD) and glutathione peroxidase (GPx) post-TBI (Hakiminia et al., 2022). Additionally, curcumin exhibits significant anti-inflammatory properties by downregulating microglial activation, reducing proinflammatory cytokines, and attenuating TLR4/MyD88/NF-κB signaling cascade after TBI (Xie et al., 2014; Zhu et al., 2014). Curcumin also reduces BBB disruption by modulating matrix metalloproteinases and tight junction proteins (Yang et al., 2007).

Curcumin shows neuroprotective effects by stabilizing mitochondrial membrane potential, inhibiting cytochrome c release and subsequent caspase-3 activation, leading to reduced apoptosis (Jayaraj et al., 2013). It also stimulates mitochondrial biogenesis in the injured rat brain by upregulating PGC-1α and NRF-1. Autophagic flux disruption is another crucial pathology in TBI mitigated by curcumin treatment via mTOR inhibition and AMPK/ULK1 pathway activation. Enhanced hippocampal neurogenesis is also evidenced upon curcumin administration in TBI models (Kim et al., 2011). Thus, the neuroprotective effects exhibited by curcumin in preclinical TBI models can be attributed to its ability to modulate oxidative stress, neuroinflammation, mitochondrial dysfunction, and autophagy. Further clinical studies substantiating curcumin’s therapeutic potential would pave the way for its clinical translation in TBI management.

### 8.4 Baicalein mitigates secondary injury after TBI

Baicalein is a bioactive flavone derived from the roots of *Scutellaria baicalensis*. Emerging evidence from preclinical studies indicates baicalein’s neuroprotective effects against neuronal damage associated with TBI. Post-traumatic seizures are a common occurrence after TBI, which exacerbates neuropathology. Baicalein administration reduces seizure severity, duration, and frequency in rodent models of TBI (Fu et al., 2020). It also restores the decreased expression of γ-aminobutyric acid (GABA) receptors induced by TBI in the rat hippocampus. Additionally, baicalein exhibits antioxidant activity by enhancing superoxide dismutase (SOD) and glutathione peroxidase (GPx) antioxidant enzymes and reducing malondialdehyde (MDA) levels after TBI (Fang et al., 2018). It also mitigates inflammation by downregulating microglial activation, NF-κB signaling, and proinflammatory cytokine production in the injured rat brain (Kong et al., 2019).

Apoptosis of neurons is a crucial contributor to TBI-induced neurologic impairments. Studies demonstrate baicalein remarkably reduces neuronal apoptosis by downregulating the expression of pro-apoptotic proteins like Bax, cleaved caspase-3, and upregulating the anti-apoptotic Bcl-2 protein (Liu et al., 2018). It also activates the PI3k/Akt pathway to inhibit neuronal apoptosis after TBI. Thus, we can conclude that baicalein treatment confers neuroprotection against post-traumatic seizures, oxidative damage, neuroinflammation and neuronal apoptosis in animal models of TBI. Further investigations into baicalein’s therapeutic potential, especially using higher-order species, would help facilitate its clinical translation for TBI management.

### 8.5 Berberine exhibits neuroprotection in TBI models

Berberine is a natural isoquinoline alkaloid found in various plants such as barberry, goldenseal, and Oregon grape. Studies indicate berberine’s therapeutic potential for ameliorating secondary brain damage following TBI. Berberine administration is evidenced to improve cognitive impairment and neurological deficits and reduce brain edema in rodent models of TBI. It is found to upregulate tight junction proteins like occludin, which helps maintain blood-brain barrier integrity after TBI (Li R. et al., 2022a). Additionally, berberine exhibits anti-inflammatory effects by inhibiting microglial activation, reducing TNF-α, IL-1β, and IL-6 levels, and attenuating the NF-κB pathway in the injured brain (Li et al., 2023a). Berberine also inhibits MMP-9 activity, thereby facilitating neuroinflammation and BBB disruption. Apoptosis of hippocampal neurons is mitigated by berberine through downregulation of ROS, increased Bcl-2/Bax ratio, and prevention of mitochondrial cytochrome c release in models of TBI. Berberine also reduces apoptosis by modulating the PI3K/Akt and p38-MAPK pathways (Han et al., 2023).

Moreover, berberine promotes neurogenesis in the dentate gyrus after TBI by increasing the expression of Brain-derived neurotrophic factor (BDNF) (Suwannakot et al., 2022). Enhanced neurogenesis by berberine leads to improved cognitive recovery following TBI. Berberine treatment confers neuroprotection against oxidative stress, neuroinflammation, and apoptosis. It ameliorates functional outcomes post-TBI. Further preclinical and clinical studies could provide evidence for berberine’s clinical utility in attenuating secondary brain injury after TBI.

### 8.6 Apigenin ameliorates neuronal damage following TBI

Apigenin is a natural flavone in many fruits and vegetables, such as parsley, onions, and oranges. Emerging evidence indicates apigenin’s therapeutic potential in attenuating secondary brain injury after TBI (Welcome, 2020). Studies demonstrate that apigenin administration improves cognitive and memory deficits in TBI rodent models, as evidenced by enhanced performance in the Morris water maze and Step-down inhibitory avoidance tests (Guo et al., 2023). Apigenin also preserves hippocampal synaptic plasticity and upregulates BDNF, contributing to its nootropic effects post-TBI. Additionally, apigenin exhibits antioxidant activity by increasing superoxide dismutase (SOD), glutathione peroxidase (GPx), and reduced malondialdehyde (MDA) levels in the injured brain (T. Zhang et al., 2015). It also stimulates Nrf2 signaling, which induces endogenous antioxidant enzyme expression after TBI.

Apigenin showed anti-inflammatory effects by suppressing microglial activation, reducing TNF-α, IL-1β and attenuating the NF-κB pathway in TBI models (Li Y. et al, 2022b). It also inhibited neuronal apoptosis by modulating Bax/Bcl-2 expression and caspase-3 activation (K. Kim and Choi, 2021). Moreover, apigenin promotes adult neurogenesis in the hippocampus, further contributing to cognitive recovery following TBI. Overall, apigenin treatment confers multifaceted neuroprotection against oxidative damage, neuroinflammation, and neuronal loss induced by TBI. Further studies could provide pre-clinical evidence supporting apigenin’s translational potential in TBI management.

### 8.7 Epigallocatechin gallate ameliorates secondary injury following TBI

Epigallocatechin gallate (EGCG) is a natural polyphenol and the most abundant catechin in green tea (M. Alam, S. Ali, et al., 2022). Emerging evidence from preclinical studies indicates therapeutic potential of EGCG in attenuating secondary brain damage after TBI (Sebastiani et al., 2021). EGCG administration significantly reduces brain infarct volume and edema in rodent models of TBI (Yessenkyzy et al., 2020). EGCG also prevents blood-brain barrier disruption by modulating tight junction proteins and restricts the extravasation of blood-derived products into the brain parenchyma. Additionally, EGCG exhibits anti-inflammatory effects by inhibiting microglial activation, reducing TNF-α, IL-1β, and IL-6, and attenuating the NF-κB pathway in the injured rat brain (Li R. et al., 2022a). EGCG also reduces neutrophil infiltration and proinflammatory cytokine production after TBI (Zeng et al., 2023). EGCG remarkably attenuates neuronal apoptosis in the hippocampus induced by TBI by upregulating Bcl-2, downregulating Bax, and cleaved caspase-3 expression (Ye et al., 2023). It also reduces apoptosis by activating the Nrf2 signaling pathway and inducing antioxidant enzymes.

EGCG promotes adult neurogenesis in the dentate gyrus and subventricular zone post-TBI, contributing to cognitive recovery (Salehpour et al., 2023). EGCG treatment confers neurovascular protection and ameliorates neuroinflammation, oxidative damage and neuronal loss induced by TBI. Further preclinical and clinical studies could provide evidence for EGCG’s translational potential in TBI management.

### 8.8 Catechin attenuates secondary pathology following TBI

Catechin is a natural flavan-3-ol found abundantly in green tea, cocoa, and many fruits. Emerging preclinical evidence indicates the therapeutic potential of catechin in ameliorating secondary brain damage after TBI (Salehpour et al., 2023). Studies demonstrate that catechin administration significantly improves neurological function, as evidenced by reduced Neurological Severity Score (NSS) in rodent models of TBI (Y. Wu and Cui, 2020). Catechin treatment also remarkably improves sensorimotor deficits post-TBI in rats (Wu and Cui, 2020). Additionally, catechin exhibits anti-inflammatory properties by suppressing microglial activation reducing TNF-α, IL-1β, and IL-6 levels. It inhibits the NF-κB pathway in the injured rat brain. Catechin attenuates neuronal apoptosis in the cortex and hippocampus induced by TBI by downregulating Bax, cleaved caspase-3 expression, and upregulating Bcl-2 levels (Gupta et al., 2021). It also reduces neuronal apoptosis by activating Nrf2 signaling and inducing antioxidant enzymes.

Catechin administration increases hippocampal neurogenesis in the subgranular zone, contributing to cognitive recovery following TBI (Z. Xu, Chen et al., 2006). Catechin treatment confers neuroprotection against neuroinflammation and oxidative damage. It improves functional outcomes post-TBI. Further studies could provide pre-clinical evidence for catechin’s translational potential in TBI management.

### 8.9 Sulforaphane mitigates secondary brain injury after TBI

Sulforaphane is an organosulfur compound derived from cruciferous vegetables like broccoli and Brussels sprouts. Emerging preclinical evidence indicates the neuroprotective effects of sulforaphane in TBI models. Studies demonstrate that sulforaphane administration improves the TBI rodent model’s cognitive function and spatial learning deficits (Gray et al., 2022). It also inhibits neuronal apoptosis in the hippocampus induced after TBI (Uddin et al., 2020). Additionally, sulforaphane exhibits antioxidant effects by increasing superoxide dismutase (SOD), glutathione peroxidase (GPx) activity, reducing malondialdehyde (MDA) and nitric oxide (NO) levels in the injured brain (Chen et al., 2021). It also induces Nrf2 signaling, which stimulates endogenous antioxidant defenses post-TBI.

Sulforaphane exerts anti-neuroinflammatory effects by suppressing microglial activation, reducing TNF-α, IL-1β, IL-6. It inhibits the NF-κB pathway in the injured cortex and hippocampus (Q. Wu et al., 2021). It also restricts the infiltration of peripheral immune cells into the brain. Sulforaphane promotes neurogenesis in the subgranular and subventricular zone post-TBI, thereby aiding cognitive recovery (Thapa et al., 2021). Sulforaphane treatment confers neuroprotection against oxidative stress neuroinflammation and improves functional outcomes in TBI models. Further clinical evaluation could provide evidence for sulforaphane’s translational value in attenuating secondary brain injury after TBI.

### 8.10 Ferulic acid exhibits neuroprotection in TBI models

Ferulic acid is a natural phenolic compound found abundantly in fruits, vegetables, and grains. Emerging evidence indicates the therapeutic potential of ferulic acid in attenuating secondary brain injury following TBI (Li Y. et al., 2022b). Studies demonstrate that ferulic acid administration dose-dependently improves neurobehavioral function, including motor coordination in mouse TBI models and reduces brain edema and neuronal loss in the injured cortex (Li R. et al., 2022a). Additionally, ferulic acid exhibits antioxidant activity by upregulating nuclear factor erythroid 2–related factor 2 (Nrf2) signaling and inducing superoxide dismutase (SOD), glutathione peroxidase (GPx) antioxidant enzymes in the injured brain (Adeyi et al., 2023). It also suppresses lipid peroxidation and nitric oxide (NO) levels post-TBI (J. Chen et al., 2023). Ferulic acid attenuates neuroinflammation by inhibiting microglial activation, reducing TNF-α, IL-1β, IL-6 levels, and NF-κB activity in the injured rat brain (Sun, Ma, Li et al., 2022). It also restricts the infiltration of peripheral neutrophils and macrophages into the brain parenchyma.

Ferulic acid inhibits the TLR4/MyD88 innate immune signaling pathway, which produces proinflammatory cytokines after TBI (Li Y. et al., 2022b). Overall, ferulic acid treatment confers neuroprotection against oxidative damage and neuroinflammation, improving outcomes in TBI models. Further clinical studies could provide evidence for ferulic acid’s translational potential for TBI management.

### 8.11 Role of limonoids in TBI

Limonoids are a class of highly oxygenated triterpenoid compounds found abundantly in citrus fruits. Emerging evidence indicates limonoids confer neuroprotection in models of neurodegenerative diseases, including TBI (Mukherjee et al., 2019). TBI pathology involves primary injury directly induced by biomechanical forces, succeeded by secondary injury mediated by excitotoxicity, calcium dyshomeostasis, oxidative stress, neuroinflammation, and mitochondrial dysfunction (Maas et al., 2017). Limonoids may confer neuroprotection by ameliorating these secondary injury factors.

Dyshomeostasis of calcium is another crucial pathological event in TBI. TBI causes excessive glutamate release and overstimulation of NMDA receptors, resulting in cytotoxic calcium influx. Limonoids may confer protection by regulating calcium flux. For instance, limonin demonstrated anti-excitotoxic effects in murine hippocampal neurons exposed to glutamate (Nagini et al., 2023). Limonin attenuated calcium influx, preventing oxidative damage and loss of viability. Mitochondrial dysfunction is both a consequence and driver of secondary injury after TBI (Thapa et al., 2021). Limonoids protect mitochondrial integrity by reducing calcium overload and oxidative damage (Qi and Wang, 2020). Limonoids may also stimulate the biogenesis of healthy mitochondria. In TBI rats, increasing mitochondrial mass, Obacunone upregulated PGC-1α and mitochondrial transcription factors.

Limonoids exhibit versatile neuroprotective mechanisms that target key secondary injury factors like neuroinflammation, oxidative stress, excitotoxicity, and mitochondrial dysfunction. While most evidence involves rodent TBI models, emerging clinical research also reveals therapeutic potential. For instance, a pilot study on TBI patients found daily orange juice consumption improved antioxidant status and reduced lipid peroxidation (Cao et al., 2021). Given the lack of effective TBI pharmacological treatments, further research on limonoids is warranted.

### 8.12 Neuroprotective role of naringenin in TBI

Naringenin is a flavonoid prevalent in citrus fruits that has exhibited antioxidant properties and shown neuroprotective effects in preclinical models of TBI (Anwar et al., 2021; Yousuf et al., 2022; Li et al., 2023b). The primary mechanical injury in TBI triggers secondary injury cascades involving neuroinflammation, oxidative stress, mitochondrial dysfunction, and excitotoxicity. Naringenin may mitigate these secondary injury factors through its anti-inflammatory, antioxidant, and anti-apoptotic properties. Naringenin reduced hippocampal expression of proinflammatory cytokines, including TNF-α, IL-1β, and IL-6, in a controlled cortical impact model of TBI in mice (Culhuac et al., 2023). This anti-neuroinflammatory effect was accompanied by attenuated neuronal apoptosis, edema, and improved cognitive function.

The anti-inflammatory actions were mediated by inhibiting NF-κB and MAPK signaling. In another study, naringenin ameliorated neurobehavioral deficits and improved long-term potentiation in the rat hippocampus after TBI by suppressing microglial activation (Wang et al., 2023). Naringenin also exhibits antioxidant activity by scavenging ROS and inducing endogenous antioxidant defenses. In a rat model, naringenin treatment following TBI elevated glutathione peroxidase and superoxide dismutase activity while reducing malondialdehyde levels in the hippocampus (Long et al., 2020). This antioxidant effect was associated with reduced neuronal apoptosis and cerebral edema. Naringenin may induce antioxidant defenses by activating the Nrf2 pathway. Mitochondrial dysfunction is a central secondary injury mechanism in TBI mediated by calcium overload and ROS-induced damage (Thapa et al., 2021).

Naringenin attenuates mitochondrial membrane potential dissipation, preserves complex I activity, and inhibits cytochrome c release in TBI rats (Bai et al., 2020). This mitochondrial protection was accompanied by reduced neuronal apoptosis. Excitotoxicity resulting from excessive glutamate release is another secondary injury factor in naringenin targets. In mixed neuronal-glial cultures exposed to glutamate excitotoxicity, naringenin inhibited calcium influx through NMDA receptors and subsequent activation of apoptotic pathways (Socała et al., 2020). Naringenin confers multifaceted protective effects in TBI models by targeting neuroinflammation, oxidative stress, mitochondrial dysfunction, and excitotoxicity. While rodent studies show promising results, clinical research is still lacking. One pilot study found that supplementation with a citrus bioflavonoid blend containing naringenin improved cognitive performance in TBI patients (Atoki et al., 2023). Further clinical trials are warranted to validate the therapeutic potential of naringenin in TBI.

## 9 Future directions and challenges

TBI has emerged as a significant global health and socioeconomic challenge, placing a substantial healthcare burden on contemporary society and necessitating the development of efficient treatment strategies (Maxwell, 2012). They suppress neuronal cell death mechanisms and restore normal function in non-neuronal cells by employing occupational therapies (Bayley et al., 2023; Wheeler and Acord-Vira, 2023). Many treatments have been devised, encompassing neurorestorative, anti-inflammatory, and neuroprotective agents.

Nevertheless, one of the immediate ramifications of TBI pertains to preserving the BBB, as the impairment of the BBB following TBI contributes to subsequent harm. Peptides and therapeutic proteins are the exclusive substances capable of traversing the endothelial tight junctions and reaching the injury site through the intranasal route. In the context of an animal model of TBI, it is possible to directly administer therapeutic agents to the cerebrospinal fluid (CSF) via an intraventricular pathway. However, in clinical practice, surgical intervention is frequently necessary to alleviate intracranial pressure and oedema, allowing for the direct delivery of drugs. Hence, the development of an efficient drug delivery system has the potential to facilitate a continuous and regulated release of therapeutic substances, thereby fostering recovery from the secondary damage to the brain following TBI (Aqel et al., 2023; Mohammed et al., 2023).

Neuroprotective approaches in treating TBI are designed to address distinct mechanisms within the intricate cascade of secondary injuries. Historically, the primary focus has been on modifying the cellular processes after the injury, constituting an essential aspect of neuroprotection. In contemporary neuroprotection strategies, the emphasis is on therapeutic interventions that initiate the restoration of neurons with optimal functionality by restraining the principal mechanism of cell death. Focusing on precise molecular mechanisms for managing and providing post-injury care in TBI is paramount. There is a critical necessity to progress and innovate new treatments to minimize the repercussions of TBI. Numerous therapies directed at specific targets have been suggested, some of which have yet to undergo clinical assessment. There should be an increased emphasis on formulating outcome-oriented treatments to address the management and treatment of TBI efficiently.

Phytochemicals have emerged as adjuvant and complementary medicines in the treatment of various disorders (Azhar et al., 2023; Majrashi et al., 2023). Phytochemicals possess various beneficial properties, including antioxidant, anti-inflammatory, anti-cancer, neuro-protectant, etc. As mentioned in the review, various phytochemicals have shown positive roles in recovery from TBI. However, various challenges are encountered in the use of polyphenols for the treatment of TBIs, which include: i. Bioavailability: Numerous phytochemicals exhibit limited bioavailability, implying they are inadequately absorbed and processed within the body, thereby constraining their capacity to reach the damaged brain tissue effectively. ii. BBB restrictions: The BBB serves as a barrier that hampers the passage of specific phytochemicals into the brain, posing a challenge for these compounds to exert their neuroprotective effects directly at the injury site. iii. Dosage: Establishing the ideal dosage and timing for administering phytochemicals to realize therapeutic advantages for TBIs is a complex task, given that distinct polyphenols can have differing pharmacokinetics and mechanisms of action. iv. Lack of clinical evidence: Although there is encouraging preclinical research indicating the neuroprotective potential of phytochemicals in TBIs, the absence of comprehensive clinical data hinders the ability to substantiate their effectiveness and safety in human patients. v. Interactions with medications: Phytochemicals can potentially interact with medications frequently employed in the treatment of TBIs, which may result in adverse effects or modifications in drug metabolism. vi. Regulatory approvals: Securing regulatory approval for TBI treatments based on phytochemicals can be a daunting task because of the intricate characteristics of polyphenols and the requirement for rigorous clinical trials.

Understanding the diverse nature of TBIs may pave the way for personalised or precision medicine strategies. Tailoring treatments to an individual’s injury characteristics, genetics, and other factors could optimize therapeutic interventions. Ongoing research into neuroprotective agents aims to identify drugs or compounds that can mitigate the initial and secondary damage caused by TBI, targeting various pathways such as inflammation, excitotoxicity, and oxidative stress. Focusing on the inflammatory response associated with TBI holds promise for future treatments, as modulating the immune response may reduce secondary injury processes and enhance overall recovery. The exploration of stem cells for neural repair and regeneration is an active area of research, offering promise for tissue healing and functional recovery after TBI.

Additionally, ongoing efforts in developing targeted drug delivery systems capable of effectively penetrating the blood-brain barrier and delivering therapeutic agents directly to the injury site represent a crucial research focus. Emerging technologies, including nanotechnology, may contribute to advancements in this field. While these future directions present potential breakthroughs, translating research findings into clinically effective treatments requires sustained dedication, collaboration, and a profound understanding of the intricate nature of traumatic brain injury (Dwyer and Morrison, 2022; Hanna and Pfister, 2023; Johnson et al., 2023; Popa et al., 2023; Rauchman et al., 2023).

## 10 Conclusion

In summary, the application of phytochemicals in the context of traumatic brain injury (TBI) presents both promising benefits and significant challenges. While preclinical studies offer robust evidence of the neuroprotective and anti-inflammatory properties associated with various phytochemical compounds, confirming their clinical efficacy and safety in human TBI patients is notably lacking, primarily due to the absence of comprehensive clinical data. A major hurdle in realizing the therapeutic potential of phytochemicals lies in their limited bioavailability and the potential for interactions with conventional medications used in TBI treatment. Determining the optimal dosage, timing, and administration methods for phytochemical interventions is complex, given the diverse range of polyphenols and their distinct pharmacokinetic profiles. Unleashing the full potential of phytochemicals in TBI management requires further dedicated research efforts. Despite the potential of phytochemicals as supplementary therapies for TBI, their integration into clinical practice and attainment of regulatory approval necessitates a thorough understanding of their impacts, a meticulous assessment of potential interactions with existing treatments, and the precise design of clinical trials. Despite these challenges, the prospective advantages of phytochemical-based treatments in improving TBI outcomes make them an enticing avenue for ongoing research and development.

## Abbreviations

Traumatic brain injury, TBI; Secondary injury cascades, SIC; Blood-brain barrier, BBB; Neurodegenerative Disorders, NDs; Alzheimer’s Disease, AD; Parkinson’s Disease, PD; Altered Levels of Consciousness, LOC; Post-Traumatic Amnesia, PTA; Glasgow Coma Scale, GCS; Computed Tomography, CT; Magnetic Resonance Imaging, MRI.
